# In the eye of the storm: SARS‐CoV‐2 infection and replication at the ocular surface?

**DOI:** 10.1002/sctm.20-0543

**Published:** 2021-03-12

**Authors:** Lyle Armstrong, Joseph Collin, Islam Mostafa, Rachel Queen, Francisco C. Figueiredo, Majlinda Lako

**Affiliations:** ^1^ Biosciences Institute, Faculty of Medical Sciences Newcastle University Newcastle UK; ^2^ Department of Ophthalmology Royal Victoria Infirmary and Newcastle University Newcastle UK

**Keywords:** ACE2, conjunctiva, cornea, coronavirus, COVID‐19, limbus, ocular surface, organ and animal models, SARS‐CoV‐2, scRNA‐Seq, tears, therapy, TMPRSS2

## Abstract

Severe acute respiratory syndrome coronavirus 2 (SARS‐CoV‐2) first emerged in December 2019 and spread quickly causing the coronavirus disease 2019 (COVID‐19) pandemic. Recent single cell RNA‐Seq analyses have shown the presence of SARS‐CoV‐2 entry factors in the human corneal, limbal, and conjunctival superficial epithelium, leading to suggestions that the human ocular surface may serve as an additional entry gateway and infection hub for SARS‐CoV‐2. In this article, we review the ocular clinical presentations of COVID‐19 and the features of the ocular surface that may underline the overall low ocular SARS‐CoV‐2 infection. We critically evaluate the studies performed in nonhuman primates, ex vivo organ culture ocular models, stem cell derived eye organoids and the differences in infection efficiency observed in different parts of human ocular surface epithelium. Finally, we highlight the additional work that needs to be carried out to understand the immune response of the ocular surface to SARS‐CoV‐2 infection, which can be translated into prophylactic treatments that may be applied to other organ systems.

## Graphical abstract

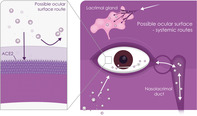

**Significance statement**

The newly discovered SARS‐CoV‐2 responsible for the COVID‐19 pandemic has been shown to affect many organs, including the lung, kidney, gut, and brain, among others. Recent work has shown that a small percentage of COVID‐19 patients present with ocular symptoms. In the present study, the authors critically evaluate the growing literature on COVID‐19 and ocular surface as well as the cell, organ, animal, and patient‐specific models used to elucidate the impact of SARS‐CoV‐2 on the ocular surface. The authors' comprehensive review suggests that ocular symptoms are most likely encountered in the initial phase of infection, with conjunctiva showing the highest propensity for infection.

## 1 INTRODUCTION

Since the discovery of yellow fever virus in 1901, more than 200 virus species that can infect humans have been discovered. Three to four new species are discovered every year,^1^ although there is an apparent slow‐down in the rate of discovery of species from different families. This century has witnessed the global spread of three previously unknown coronaviruses. In November 2002, the first case of severe acute respiratory syndrome (SARS) was reported in China. By July 2003, 8096 reported cases including 774 deaths in 27 countries were documented.^2^ Ten years later, a novel coronavirus, named Middle East respiratory syndrome (MERS) was isolated, causing infection of 1728 confirmed cases, including 624 deaths in 27 countries.^3^ In December 2019, new unexplained cases of pneumonia and respiratory distress were reported by officials in Wuhan, China.^4^ This was soon followed by reports of clusters of cases within families^5^, ^6^ and infections of healthcare workers,^7^ which indicated human‐to‐human transmission. In January 2020, the pathogen named severe acute respiratory syndrome coronavirus 2 (SARS‐CoV‐2), belonging to the family of coronaviruses was identified. The virus spread quickly in both hemispheres and the World Health Organization (WHO) declared coronavirus disease 2019 (COVID‐19) a public health emergency on the 30th January 2020 and subsequently a pandemic on 11th March 2020. As per 25th January 2021, 97 831 595 confirmed cases, including 2 120 877 deaths were reported globally.

**FIGURE 1:**
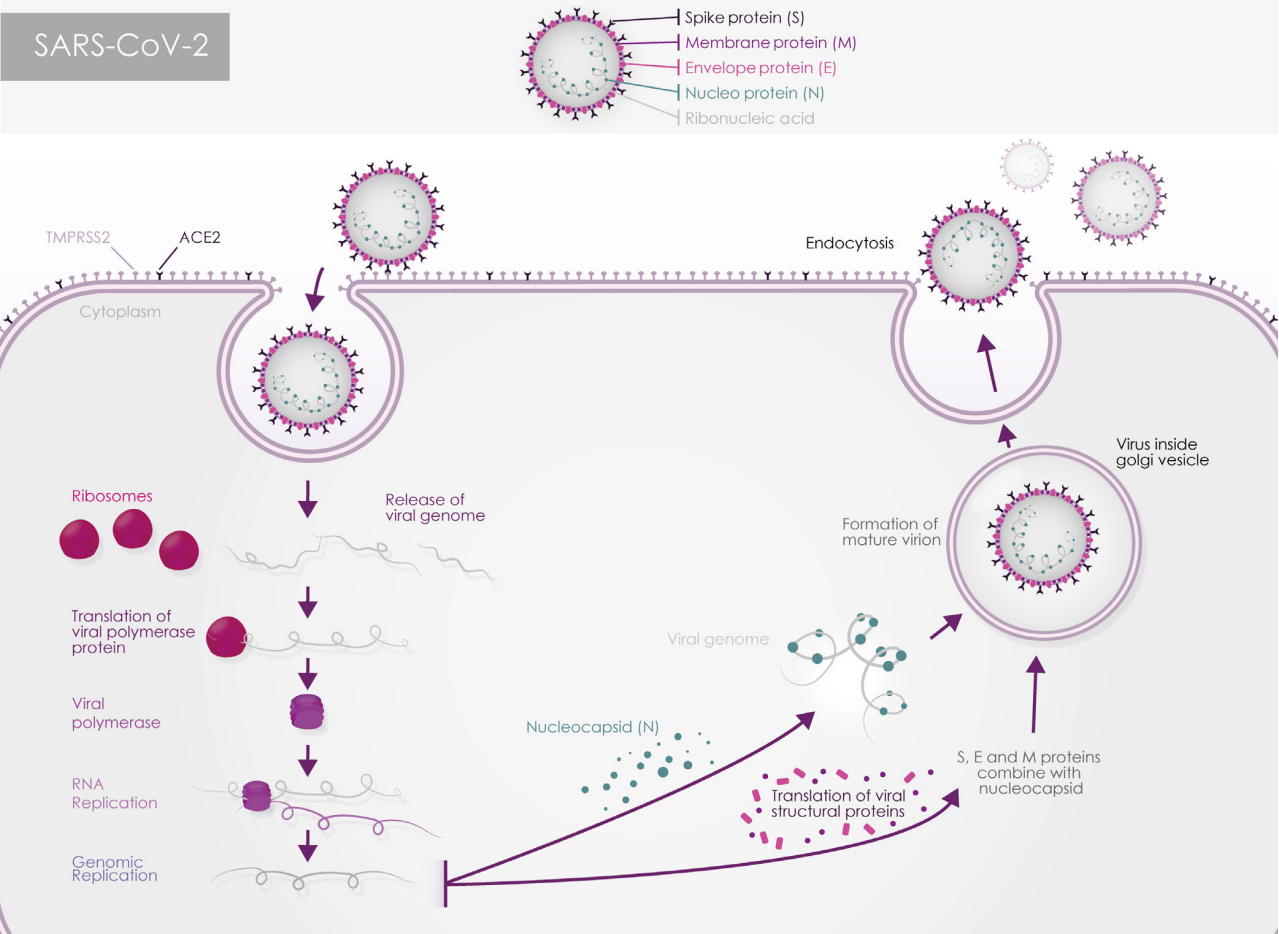
Schematic presentation showing the viral entry and propagation into the host cell

Coronaviruses (CoVs) belong to the Coronaviridae family of order Nidovirales. They are classified into four genera: Alpha‐, Beta‐, Gamma‐ or Delta‐coronavirus. The CoVs genome is 26‐32 kb long, consisting of enveloped, single stranded positive‐sense RNA that is 5′ capped and 3′ polyadenylated.^8^ This is the largest RNA genome reported to date.^9^ The CoV particles are spherical in shape with 50‐200 nm diameter. In accordance with their parasitic nature, CoVs like many other viruses rely on host cells to complete their life cycle (Figure 1). Following entry into the cells, the viral genome RNA is translated to generate all the proteins needed for RNA replication and transcription, a process, which requires involvement of several host proteins.^8^

The CoVs RNA genomes are characterized by the presence of two large open reading frames (ORFs) 1a and 1b, which comprise more than 2/3 of the genome and encode 23 putative proteins including a 5′‐replicase, spike (S), envelope (E), membrane (M), and nucleocapsid (N)‐3′ proteins (Figure 1). The structural proteins S, E, M, and N are common to all known coronaviruses.^10^ They function during host cell entry, viral morphogenesis, and virion release. The S protein is a membranous glycoprotein, which is essential for viral entry and defining host range, tissue tropism and virulence. M protein is localized at intracellular membrane structures and facilitates the change in the shape of the viral particle and its attachment to the nucleocapsid. The envelope protein (E) supports the manufacture and emission of particles. Lastly, the nucleocapsid protein (N) supports the replication of CoV genes by helping attach the genome to the replication‐transcription complex. The interaction between the M and E proteins and nucleocapsid results in budding through the membrane (Figure 1).

SARS‐CoV‐2 is a novel enveloped RNA Betacoronavirus2, which results in COVID‐19 infectious disease and primarily affects respiratory tissues, although increasing evidence indicates that multiple other organs including heart,^11^ kidneys,^12^ gut,^13^ liver,^14^ and the neurological system^15^ may be affected. SARS‐CoV‐2 is transmitted through zoonosis with a primary reproduction number of approximately 2.7, which indicates an exponential growth rate of infections. For symptomatic patients, clinical prognosis can be divided into three patterns: mild illness with upper respiratory tract symptoms, nonlife‐threatening pneumonia, or severe pneumonia with acute respiratory distress syndrome (ARDS) that begins with mild symptoms for around a week, then progresses to necessitate advanced life support.^16^

The newly sequenced SARS‐CoV‐2 genome is ~29.9 kb and shares 82% identity with SARS‐ and MERS‐CoV.^17^ Phylogenetic analyses of 160 human SARS‐CoV‐2 genomes performed in March 2020 indicated three central variants, named A, B and C, which differ from each other by few amino acid changes.^18^ A subsequent study of 95 human SARS‐CoV‐2 genomes in April 2020 identified 116 mutations,^19^ indicating that the pool of circulating viruses in the early phases of the COVID‐19 pandemic was highly diverse with some strains leading to greater transmission than others. Intense global efforts are focusing on sequencing a large number of SARS‐CoV‐2 strains to facilitate a detailed understanding of viral mutations and transmissions and provide valuable insights into vaccine development and resistance against antiviral drugs.^20^

Recent clinical evidence has suggested that COVID‐19 may present with ocular symptoms as well as pathological changes in the ocular surface in a minority of patients.^21^, ^22^, ^23^ Importantly, work published earlier this year by our group and others has shown the presence of SARS‐CoV‐2 entry factors ACE2 (angiotensin‐converting enzyme 2) and TMPRSS2 (Transmembrane Serine Protease 2) in the superficial corneal, limbal and conjunctival epithelium, which led to suggestions of ocular surface serving as an additional entry gateway and potentially a replicative hub.^24^, ^25^

In this article, we review the COVID‐19 ocular clinical presentations and the features of ocular surface, that may underline the overall low ocular SARS‐CoV‐2 infection. We critically evaluate the studies performed in nonhuman primates, ex vivo organ culture ocular models, stem cell derived eye organoids and the differences in infection efficiency observed in different parts of human ocular surface epithelium. Finally, we highlight the additional work that needs to be carried out to understand the immune response of the ocular surface to SARS‐CoV‐2 infection, which can be translated into novel prophylactic treatments that may be applied to other organ systems.

## 2 COVID‐19 AND OCULAR MANIFESTATIONS

The first report of ocular manifestation among COVID‐19 patients was provided by a member of the national expert panel on pneumonia, who was infected during his inspection in Wuhan, despite wearing an N95 mask.^26^ This expert did not wear an eye protection or face shield and several days prior to developing pneumonia, he reported redness of his eyes, which led to the suggestion that unprotected exposure of the eyes might have allowed the virus to infect the body.^26^

In a case‐series, 38 COVID‐19 positive patients treated at a hospital in Hubei Province, China were retrospectively reviewed for ocular symptoms.^23^ Of these patients, 31.6% had manifestations consistent with conjunctivitis, such as epiphora, chemosis, conjunctival hyperemia, or increased secretions. The study also reported that patients with ocular symptoms were more likely to have higher white blood cell, neutrophil counts, procalcitonin, C‐reactive protein as well as lactate dehydrogenase than patients without ocular symptoms, which suggest that ocular symptoms were more likely to appear in patients with severe pneumonia.^23^ However, limitations of this study were the relatively small sample size, sampling of one eye only and absence of detailed ocular examinations to exclude other ocular diseases. A larger study of confirmed COVID‐19 patients indicated a range of ocular symptoms including itching, redness, tearing, discharge and foreign body sensation; however, only 3 out of the 121 patients yielded positive results from the conjunctival swabs and only one of these was among the eight identified as having ocular symptoms.^21^ A low frequency of conjunctival congestion (0.8%) was also reported in the largest retrospective study of 1099 confirmed COVID‐19 patients that were hospitalized in 552 hospitals across 30 provinces in China.^27^ Similarly, a low frequency of viral RNA detection in tears and conjunctival secretions has been reported^28^ 3‐7 days since disease onset (Table S1), which suggests that ocular surface infections may be predominant in the early phase of disease. Notwithstanding, isolated case studies have described the virus persistence in the ocular surface past the initial infection phase. For example, a study performed by Chen et al indicated the development of bilateral acute follicular conjunctivitis at day 13 of illness in a 30 year old COVID‐19 patient, with SARS‐CoV‐2 RNA being present in the conjunctival specimens between 9 and 18 days of disease.^29^ Prolonged presence of viral RNA has also been described in a clinical case report of a Chinese COVID‐19 positive patient who traveled from China to Italy and presented with bilateral conjunctivitis at day 1 of hospitalization. Viral RNA was detected by RT‐PCR on the conjunctival swab samples from day 3 to day 21 at lower Ct values than nasal swabs. While no viral RNA was detected between days 22 and 26 in both nasal and conjunctival swabs, low expression was detected in conjunctival swabs at day 27, which indicates a sustained infection, also corroborated by the successful viral inoculation in Vero E6 cells.^28^

To ascertain the impacts of COVID‐19 on the ocular surface, a prospective observational study assessed 38 confirmed COVID‐19 patients and 31 healthy controls.^30^ While no significant differences were observed regarding age and gender between the two groups, conjunctival impression cytology revealed decreased density and enlargement of goblet cells, squamous changes, and increased presence of neutrophils in the COVID‐19 patients. Together these data demonstrate that pathological alterations may be present in the ocular surface at the beginning of COVID‐19 without significant ocular manifestations. Importantly, the neurological impact on vision has not been fully addressed. A retrospective study of the clinical manifestations of 214 patients with COVID‐19 in three hospitals in Wuhan reported that 1.4% (3/214) had visual impairment.^31^ The study did not elaborate on the nature of those visual impairments.^31^ There were also two reports of patients diagnosed with COVID‐19 after presenting with diplopia and ophthalmoparesis from cranial nerve palsy.^32^

Overall, the frequency of viral detection in tears and conjunctival sample of COVID‐19 patients published to date is low and mostly in the initial phase of the infection. Furthermore, when the ocular symptoms were observed, conjunctivitis seems to be the most common ophthalmologic sign related to coronavirus infection.

## 3 HUMAN OCULAR SURFACE AND SUSCEPTIBILITY TO SARS‐CoV‐2 INFECTION

### 3.1 The ocular surface

**FIGURE 2:**
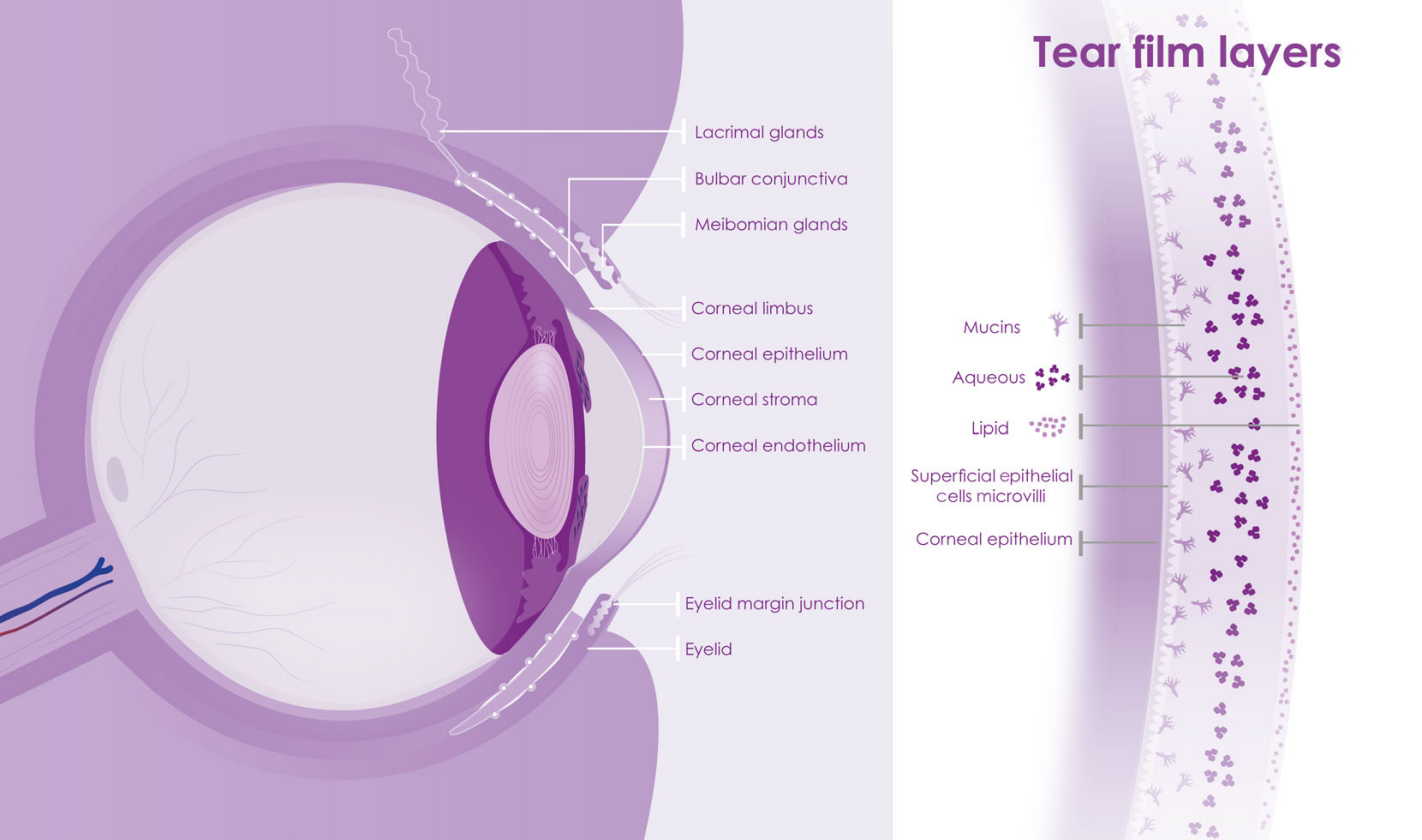
Schematic presentation showing the ocular surface consisting of cornea, conjunctiva, and tear film on the left and tear film layers (superficial lipid layer, aqueous component, and mucins) on the right

The ocular surface is commonly described as a continuous epithelium of the cornea, conjunctiva, and associated glands, which exudes and is bathed in the tear film (Figure 2). All these components are required to act in concert to maintain a hydrated and protective barrier for the exposed surface of the eye. The tear film acts as the first line of defense, is complex and contains three layers secreted by different glands and tissues, that is, a superficial lipid layer, an aqueous component, and mucins. The lipid‐rich secretion, which prevents evaporation of the aqueous middle layer, is secreted by the meibomian glands of the eyelids.^33^ The lacrimal and accessory glands provide an aqueous lacrimal fluid of water, proteins, and electrolytes. The cornea and conjunctiva epithelial cells, conjunctival goblet cells and lacrimal gland produce the mucins and electrolytes, which constitute the innermost mucous layer of the tear film.^34^, ^35^ Transmembrane and soluble mucins interact to form the glycocalyx layer on top of the epithelia. The cornea and conjunctiva epithelia, through tight junctions, form a physical barrier and substrate for the tear film. And microvilli help anchor the tear film to the epithelia.^36^, ^37^

An integral element of the ocular surface functional unit is the lacrimal system. This has been described to include the main and accessory lacrimal glands, meibomian gland, cornea, limbus, conjunctiva, tear film, eyelids, nasolacrimal drainage duct, and interconnecting innervation.^38^ The lacrimal gland itself not only secrets the aqueous tears (including water, electrolytes, protein, and mucus) maintaining a moist environment, but also secretes the microbicidal proteins lysozyme, lactoferrin, phospholipase, lacritin, immunoglobulins and cytokines, and via plasma cells, IgA to help protect against pathogens.^39^ The tear film is drained via the efferent tear ducts through the nasal lacrimal passages formed of the lacrimal canaliculi, and sac, and nasolacrimal duct, linking the tear duct to the nasal passages, and into the surrounding cavernous vascular system. These passages are lined by epithelium with most epithelial cells presenting microvilli and some reports of motile cilia. Goblet cells reside within this epithelium, sometimes grouping to form mucous glands.^40^, ^41^ Similar to the cornea and conjunctival epithelial cells, conjunctival goblet cells and lacrimal gland,^34^, ^35^ the lacrimal sac and nasolacrimal duct secrete mucins,^42^, ^43^ providing a similar epithelial environment to the ocular surface (and other mucosal epithelia throughout the body) that may be infected by viruses.

### 3.2 COVID‐19, the ocular surface, and ocular‐systemic transmission

**FIGURE 3:**
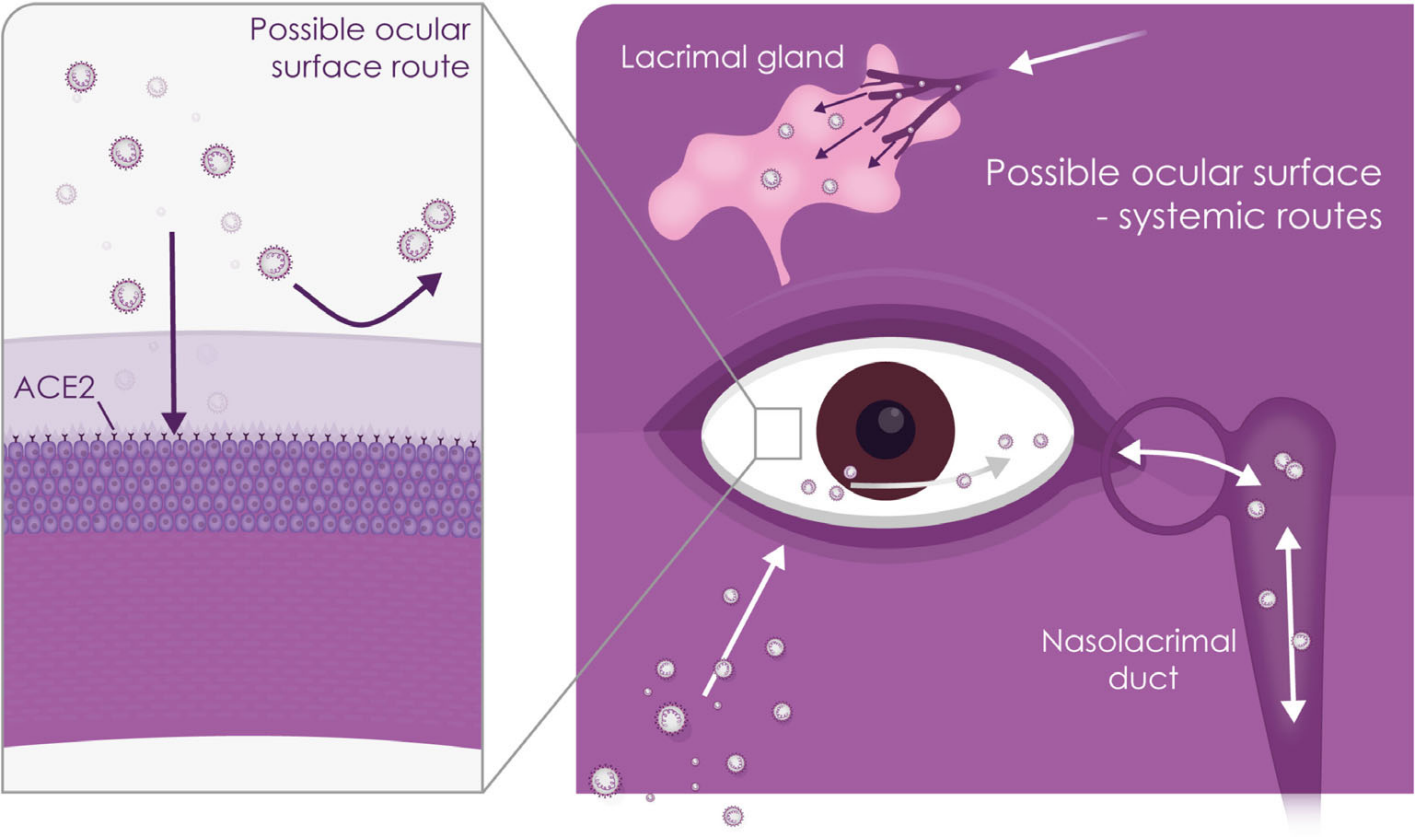
Schematic showing potential SARS‐CoV‐2 entry into the eye via infection of ocular surface epithelium (left hand side panel) or the systemic routes (right hand side panel)

As summarized above, SARS‐CoV‐2 infection of the ocular surface is observed at low frequency.^44^ Currently the potential of the ocular surface as a mucous membrane to act as a site for infection, replication and transport of SARS‐CoV‐2 remains to be elucidated. As discussed, corneal and conjunctival epithelial cells with the appropriate receptor and protease may be able to act as a direct inoculation site from infected respiratory droplets (Figure 3). However, the tear film and the fast drainage (approximately every 5 minutes) may provide a barrier for infection of the underlying epithelia. This may explain the scarce reports of conjunctivitis and absence of keratitis among COVID‐19 infected patients. It has been postulated that the tear film, particularly the superficial lipid layer, may act as a barrier to prevent SARS‐CoV‐2 binding to the corneal/conjunctival epithelia entry receptors. Furthermore, the tear flow may provide an “ocular surface wash out” effect, preventing prolonged persistence of virus on the ocular surface. Nonetheless, if the virus makes its way to the ocular surface epithelium, through the tear film, then the latter through its lipid nature (and thus adherence of virus), tear flow and drainage may facilitate a second route of infection binding to receptors in and beyond the nasolacrimal system.^45^, ^46^ In addition, the conjunctival epithelium is known to secrete mucins that contain sialic acids,^47^ which promote infection by the porcine CoV transmissible gastroenteritis virus (TGEV) under unfavorable environmental conditions.^48^ And with studies showing that many viruses have been detected in tear fluids and conjunctival swabs,^49^, ^50^ including SARS‐CoV‐2,^23^, ^51^ the possibility of viral transmission warrants further investigation. Furthermore, this route of infection has been tested in primates (see Section 3.4) resulting in a mild respiratory infection.^46^ The rarity of conjunctivitis^22^, ^28^, ^29^ and the infection of conjunctival explants^52^ provide some evidence that the tear film is protective.

The role of the nasolacrimal ducts in linking the nasal cavity‐associated lymphoid tissue with the ocular mucosal immune system contributes to the immunological interdependence between the ocular and respiratory systems.^47^, ^53^ This may be relevant given recent findings showing expression of SARS‐CoV‐2 entry genes in nasal epithelial cells together with innate immune genes.^54^ The authors suggest the nasal epithelial cells may therefore have a role in viral infection, spread and clearance.^54^ There have also been suggestions that cellular receptors in the system linking the ocular and respiratory systems may affect the tissue tropism of respiratory viruses, and proposals that mechanisms may exist which confer an ocular tropism to some viruses.^47^

Thus, the nasolacrimal system as a route for migration of the virus, either through drainage of tears through the duct to the respiratory tract or from the upper respiratory tract through the nasolacrimal duct to the eye may provide an additional route of entry and infection of other tissues including the epithelium of the lacrimal, nasolacrimal drainage system, nasal passage, and upper respiratory tract.^55^, ^56^ Furthermore, hematogenous infection of or from the lacrimal gland or duct may also be a route worthy of investigation,^56^ although to date there has not been a single report of dacryoadenitis or lacrimal gland infection, which makes this mechanism of infection less likely.

### 3.3 The expression of SARS‐CoV‐2 entry and restrictive factors in the human ocular surface

For the CoV genome to enter the host cell, the S1 unit of the spike protein needs to bind to a cellular receptor at the host cell surface (Figure 1). Work published this year, indicated that the angiotensin converting enzymes 2 (ACE2) acts as a receptor for SARS‐CoV‐2.^25^, ^57^, ^58^ In addition, viral entry also requires S priming by host proteases, which enable fusion of the viral and cellular membranes.^59^ S priming is carried out by transmembrane protease serine type 2 (TMPRSS2). In the absence of proteases in the host cell surface, the virus can enter the cell through an endosomal pathway, which is at least 100 times less efficient than the primary route.^60^ In this pathway, the S protein is instead activated by the endosomal cathepsin B/L.^60^ A recent non‐peer reviewed study, also suggests binding of the S protein to the CD147 cell surface receptor as a novel entry route for SARS‐CoV‐2 in human cells.^61^ Accordingly, a recent clinical trial employing a humanized anti‐CD147 antibody has shown a significant improvement in the case severity of critically ill patients.^62^ Interestingly, CD147 is expressed on the cell surface of human corneal epithelium, stroma and endothelium and conjunctival epithelium.^63^

Many epithelial tissues as well as various eye compartments (Table S2) have been shown to express ACE2 and TMPRSS2. In a recent study, 38 conjunctival samples from 8 healthy subjects and 30 patients were analyzed with bulk RNA‐Seq sequencing for the expression of ACE2 and TMPSS2.^64^ The authors reported that both ACE2 and TMPRSS2 mRNA and protein were not substantially expressed in the conjunctival samples and thus suggested that COVID‐19 transmission via this pathway would be unlikely.^64^ One limitation of this study is that 68% of the samples were collected from patients, with either melanomas, squamous cell carcinomas or papilloma, and so may not reflect the conjunctiva of most patients with COVID‐19. Furthermore, the bulk RNA‐Seq can only reflect the average gene expression; hence, if the SARS‐CoV‐2 entry genes were expressed in a relatively small fraction of cells in the conjunctival epithelium, they would be missed with this approach. These technical issues could also be an underlying factor for the findings presented by Ma and colleagues, who reported consistent expression of *ACE2* in two out of three primary human conjunctival samples, but no detectable *TMPRSS2* using RT‐PCR.^65^

In an effort to investigate at the single cell level the expression of ACE2 and TMPRSS2, single cell RNA‐Seq studies have been performed indicating high *ACE2* and *TMPRSS2* expression in the upper respiratory system (nasal epithelial cells) and at lower level of expression in the superficial conjunctival epithelium.^54^ In a subsequent study focused only on the human ocular surface, two groups including ours, reported co‐expression of *ACE2* and *TMPRSS2* in the conjunctival, corneal and limbal epithelium^24^, ^25^ alongside a ubiquitous viral receptor heparin sulfate, which can facilitate the initial viral attachment.^66^ The expression of ACE2 and TMPRSS2 in the superficial conjunctival and corneal epithelial surface was also corroborated by immunohistochemistry and Western blotting.^24^, ^25^ Importantly, we identified TNF, NFKβ and IFNG as upstream transcriptional regulators of ACE2 and TMPRSS2 in the superficial conjunctival epithelium, suggesting that pro‐inflammatory signals may fine‐tune the response of ocular surface epithelium to SARS‐CoV‐2 infection.^24^ The presence of SARS‐CoV‐2 entry factors on the ocular surface is not the ultimate arbiter of infectability since the percentage of cells that express both entry factors may enhance or suppress viral entry into the eye. Our single cell RNA‐Seq study published earlier this year, indicated that only 6.6% of cells of the superficial conjunctival epithelium express both entry proteins^24^; hence, questions remain whether this is sufficient to enable robust SARS‐CoV‐2 entry into the ocular surface and moreover establish a productive infection.

**FIGURE 4:**
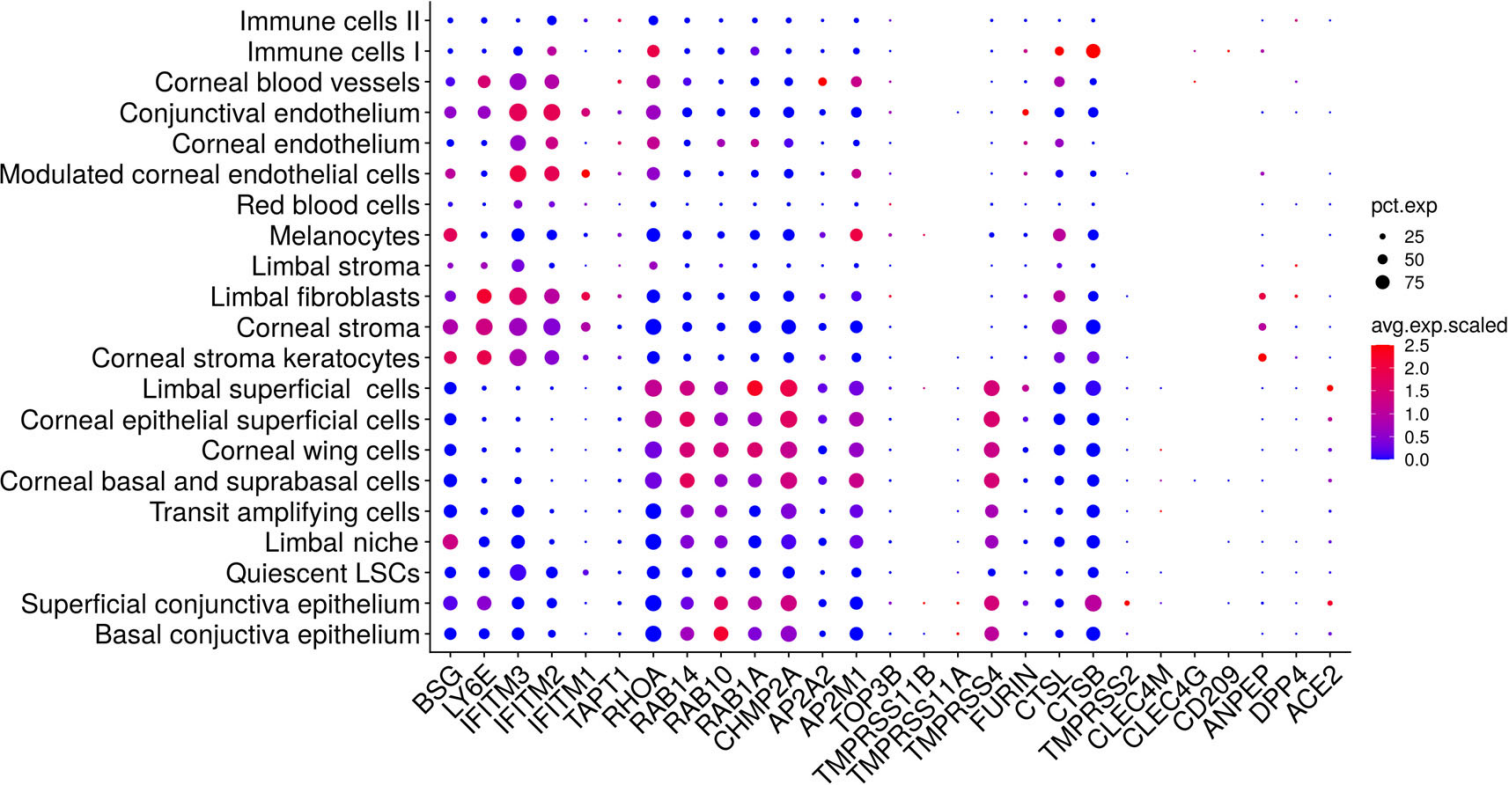
Expression of coronavirus‐associated receptors and factors in human ocular surface. Single cell RNA‐Seq of human cornea was performed and the expression of coronavirus‐associated receptors and factors was assessed and shown as dot plots. The size of the dots indicates the proportion of cells, while the color indicates the mean expression

Recent papers have also implicated other receptors and proteases that may mediate human CoV entry and possibly SARS‐CoV‐2, acting alongside ACE2 receptor and TMPRSS2 protease. These include CD147 (BSG), CD13 (ANPEP), CD209 (CLEC4L), CLEC4GL, CD299 (CLEC4M) receptors and TMPRSS4/11A/11B proteases. A detailed analysis of their expression using the single cell RNA‐Seq data obtained by our group, indicates that *CD147 (BSG)*, *CD13 (ANPEP)*, *CD209 (CLEC4L)*, *CLEC4GL*, and *CD299 (CLEC4M)* are either absent or expressed at very low levels and thus unlikely to play a major role in SARS‐CoV‐2 infection of ocular surface (Figure 4). On the contrary, expression of *TMPRSS4* is high in the human ocular surface epithelium, which may suggest a joint function between TMPRSS2 and TMPRSS4 in promoting SARS‐CoV‐2 entry as shown recently in the human small intestinal enterocytes.^67^

Once the virus is able to enter the cells, it also requires host cell proteins for genome replication (*TOP3B*, *MADP1*), viral traffic and/or assembly (*AP2M1*, *AP2A2*, *CHMP2A*, *RAB1A*, *RAB10*, *RAB14*, *RHOA*, *TAPT1*).^68^ A re‐examination of our single cell RNA‐Seq data of the ocular surface^24^ indicated that host cell genes, which mediate viral traffic and/or assembly are expressed in all part of the superficial ocular surface; however, the host genes required for genome replication are either absent (*MADP1*) or expressed at very low level (*TOP3B*), which may suggest that although SARS‐CoV‐2 can enter the ocular surface it may not be able to replicate productively (Figure 4). Importantly, *LY6E*, a restrictive factor, is also expressed in the superficial ocular conjunctival epithelium, suggesting the presence of some restrictive response for the virus in the ocular surface. These data are all at the transcriptional level and a lot more work is required to investigate their expression at protein level in a large number of people at different age and gender as well as their role in mediating SARS‐CoV‐2 infection and replication in the ocular surface. It would also be of great interest to examine whether topical eye drops, wearing spectacles, contact lenses wear or ocular surface diseases such as dry eye also have an impact on the expression of SARS‐CoV‐2 entry and processing factors in the ocular surface.

Apart from expression of entry and restrictive genes, we should be mindful about the expression of proteins involved in innate immune response, which are present in the tears. A prominent example is lactoferrin, an iron‐binding glycoprotein, representing 25% of total tear proteins. Lactoferrin is known for its antiviral activity, which is exerted through binding to cell surface proteoglycans as well as binding to viral particles themselves and upregulation of the IFNα and enhancement of the NK and T cell activity.^69^ Published evidence suggests that lactoferrin prevents SARS‐CoV binding to heparan sulfate proteoglycans.^70^ If this was to prove true also for SARS‐CoV‐2, lactoferrin would provide a potential prophylactic solution for exposed surfaces such as the ocular, buccal, and nasal epithelium.

### 3.4 Can SARS‐CoV‐2 infect the ocular surface?

The study of animal models that recapitulate human disease is an essential prerequisite for understanding the pathology of infection via the ocular surface. Nonhuman primates have been used to investigate the mechanisms of SARS‐CoV and MERS‐CoV infections. Cynomolgus macaques infected with SARS‐CoV via the conjunctival route demonstrated viral replication and developed neutralizing antibodies at 8‐week post infection. Their chest symptoms peaked between day 8 and 10 of infection and overall, infection by the conjunctiva was more efficient than intravenous route.^71^ Viral RNA was detected in the nasal and throat swabs from day 0 to 2 post infection, suggesting viral transfer from the ocular surface to respiratory tract and other tissues.

Rhesus macaques were also infected with MERS‐CoV using a combination of intratracheal, intranasal, oral, and ocular surface routes.^72^ The virus could be detected in conjunctival swabs by 3 days post infection, but it was no longer present by day 6, indicating that conjunctival testing should be done relatively early during the disease.

Following on a similar path, Deng and colleagues infected three Rhesus macaques with SARS‐CoV‐2: two via the ocular conjunctiva inoculation and one via the intratracheal route.^46^ The viral RNA could be detected in the conjunctival swabs 1 day after infection in the animals infected via the conjunctival route; however, no histology was performed on the eye and no signs of inflammation, conjunctivitis or keratitis were reported. In contrary, viral RNA was detected in nose swabs and throats of animals inoculated via the ocular surface from day 1 to 7 post infection, indicating that the inoculated SARS‐CoV‐2 could have transferred from the ocular surface to the respiratory tract from day 1 onwards. This was corroborated by the postmortem analysis, which revealed viral load distribution in the ocular tissue, but also in the nasolacrimal system, nose, pharynx, and trachea as well as other tissues including the lung, stomach, duodenum, caecum, and ileum. The viral load detected in the lung was lower in the macaques injected via the conjunctival compared with tracheal route, which indicates that in these animals the most likely route of infection was via the ocular surface; however, this cannot be separated from inoculation via other routes (eg, nasal epithelium). The authors also showed that monkeys infected via the tracheal route displayed weight loss; however, this was not the case for the animals infected via the ocular surface. This led the authors to suggest that conjunctival inoculation resulted in a mild lung infection compared with the trachea inoculation.

To investigate whether the conjunctiva may act as an entry portal for SARS‐CoV‐2, human conjunctival and corneal explants were infected with SARS‐CoV‐2 in two recent studies. In the first study, Hui et al, found evidence of infection and productive replication in the human conjunctiva explants^52^; moreover, the conjunctival explants were more effectively infected with SARS‐CoV‐2 than by SARS‐CoV. The study used a small number of donors and could not reproduce the effect of tear flow in washing the virus out of the ocular surface; hence these results need to be interpreted with caution until further data become available. In the second study, Miner et al showed that human corneal explants were not permissive to SARS‐CoV‐2 replication in seven donors, even after the blockade of the type III interferon (IFN) receptor, which is known to limit Herpes Simplex virus 1 (HSV‐1) and Zika virus growth in the human cornea.^73^ It is not clear, if the differences in outcomes between these two studies reflect the different susceptibility of conjunctival and corneal epithelium to SARS‐CoV‐2 infection and replication and thus further work using both types of explants as well as in vitro models of conjunctival and corneal epithelial surface is needed. In addition, work with pathological specimens is also necessary to understand the SARS‐CoV‐2 tropism in the human ocular surface. A recent study of 10 confirmed COVID‐19 deceased individuals (median age 66, range 46‐90 years old) indicated the positivity rate for SARS‐CoV‐2 to be 15% for conjunctiva, 5% for the anterior corneal surface and 15% for the vitreous.^74^ In summary, the limited number of studies published to date indicate that SARS‐Cov‐2 may be able to infect the conjunctival epithelium, although the infection via the ocular surface route may result in a mild lung infection compared with inoculation via the trachea. The concept of ocular surface infection is important not only for better prevention, detection, and treatment of COVID‐19 patients, but also for testing the ocular tissues intended for transplantation. The above recent study performed by Sawant et al reported that of the 132 ocular tissues from 33‐surgical donors, positivity rate for SARS‐CoV2 was ~13% (17/172).^74^ The viral spike and envelope proteins were detected in the epithelial layers of the corneas that were procured without povidone‐Iodine disinfection, strongly suggesting that the latter should become a norm for eye banks supplying corneas for transplantation purposes.

## 4 STEM CELL MODELS FOR UNDERSTANDING SARS‐CoV‐2 ENTRY AND/OR REPLICATION IN THE HUMAN OCULAR SURFACE

Given the impact of COVID‐19 on human health, the development of therapies directed against the virus itself or against the physiological consequences of SARS‐CoV‐2 infection, is an urgent and unmet need. Numerous academic and commercial organizations are engaged in the search for both novel and re‐purposed small molecule antiviral therapies, vaccines, anti‐inflammatory agents, convalescent plasma infusions, natural killer cells and speculative cell therapy approaches.^75^, ^76^, ^77^ To enable and inform these worldwide efforts, a thorough understanding of SARS‐CoV‐2 tropism needs to be gathered systematically in different tissues and organ systems. Tissues obtained from COVID‐19 infected patients have been widely used to understand the pathology of the disease; however, in most cases these represent the end stage of infection and do not permit multiple experimental questions to be investigated in detail. Hence, lab made tissue models which can be generated with ease are needed to obtain new insights into SARS‐CoV‐2 infection and the response of each tissue to the infection. Both adult and pluripotent stem cells differentiated to tissue specific lineages are being used to generate organoids of different types (brain, lung, gut, kidney, etc)^78^ and cell monolayers (eg, pancreatic beta cells).^79^ In this section we review how stem cells are and can be used to understand how SARS‐CoV‐2 infects various parts of the ocular surface.

A whole eye organoid approach from human pluripotent stem cells containing retina, retinal pigment epithelium, ciliary margin, lens, cornea and iris as well as cell monolayers grown from limbal, corneal and conjunctival epithelium were recently used to understand effectiveness of SARS‐CoV‐2 infection in the ocular surface.^80^ The authors of this study found that limbus was the most susceptible to infections, with central cornea exhibiting very low levels of viral replication, which corroborates previous findings of corneas being refractory to infection.^73^ Furthermore, the authors concluded that even though type I and III interferons were not detected, human eye organoids mounted an immune response indicative of the NFKB mediated pro‐inflammatory cytokine response. Notwithstanding these interesting findings, the cell differentiation approaches used for the generation of human eye organoids resulted in a mix of epithelial cells from various parts of the ocular surface. Furthermore, the cell monolayers grown from the limbal, corneal, and conjunctival epithelium were not subjected to a directed differentiation protocol or analyzed in detail to assess whether they represented a basal or superficial epithelium, which may complicate data interpretation. As an alternative, we suggest two additional approaches for generating cells of ocular surface as follows: (a) directed differentiation of human pluripotent stem cells to ocular surface epithelial cells and (b) air liquid interface differentiations of ex vivo expanded epithelial stem cells from different parts of the ocular surface. Our group has reported in the recent years a robust differentiation protocol, which combines bone morphogenetic protein 4 (BMP4), all trans‐retinoic acid and epidermal growth factor for the first 9 days of differentiation followed by cell re‐plating on collagen‐IV‐coated surfaces with a corneal‐specific‐epithelial cell media for an additional 11 days, resulting in step wise differentiation of human embryonic stem cells (hESC) to corneal epithelial progenitors and mature corneal epithelial‐like cells.^81^ The relatively short differentiation protocol and the ability to generate many mature corneal‐epithelial like cells, makes this protocol suitable for infection and testing of a range of broad‐spectrum antivirals in existing topical ophthalmic medications. To date, similar protocols have not been generated for the differentiation of human pluripotent stem cells to conjunctival epithelium; hence, to generate these as well as limbal epithelium, air liquid interface differentiations of basal cells expanded from the limbal,^82^ conjunctival,^83^ and corneal epithelium^84^ can be carried out. The advantage of these air liquid interface differentiations is that cells of the superficial ocular surface epithelium can easily be generated within 2 weeks of differentiation (unpublished data from our group), exhibiting high expression of SARS‐CoV‐2 entry factors and the typical cytokeratin and mucin expression associated with different regions of the ocular surface epithelium, enabling direct investigations into the entry mode for SARS‐CoV‐2, the ability to establish a productive replication and immune response mounted by these cells. This model may also address questions about the role of additional entry factors (eg, other member of the TMPRSS family, furins, etc) and testing of therapies to ascertain whether SARS‐CoV‐2 ocular entry and/or propagation in the ocular surface can be inhibited. For example, by application of antisense oligonucleotides, to knockdown expression of key components of the viral envelope, or the addition of human recombinant ACE2 protein to inhibit viral entry. The advantage of the ocular surface is that most of the above therapies, if successful, can be easily translated to “eye drop” treatments in view of the existing knowledge of topical ophthalmic medications.

## 5 SUMMARY AND FUTURE DIRECTIONS

In this review, we have shown that the incidence of associated ocular signs and SARS‐CoV‐2 detection in tears and conjunctival samples to date is relatively low and mostly observed in the initial phases of COVID‐19 infection. We have also evaluated the work performed in explant and cell models, which show that conjunctival and limbal epithelium are infected by SARS‐CoV‐2, but the corneal epithelium is refractory to the infection. This low detection of SARS‐CoV‐2 in the ocular surface compared with respiratory system may be due to the open ocular surface environment, which can wash the virus away via tears, transport the virus to the nose rapidly, and/or the immune surveillance developed by the ocular surface, that might render the ocular surface less susceptible to SARS‐CoV‐2 infection. The low detection rate of SARS‐CoV‐2 in the ocular surface may also be due to technical issues related to the small sample volume of tears and ocular secretions, which may yield low RNA amounts for accurate viral detection, variable sampling times and frequency during disease progression, difference in methods and tools used for ocular sample collection and relatively small patient numbers in most studies. It is also possible that widespread usage of ophthalmic medications (eg, artificial tears, topical glaucoma medications) may represent a potential form of treatment for SARS‐CoV‐2 replication or diffusion in the ocular surface, thus reducing the incidence of conjunctivitis in COVID‐19 patients.^85^ Most importantly, we must not forget that not all patients with conjunctivitis, but without typical symptoms of COVID‐19 are clinically evaluated; hence, it is possible that both symptomatic or asymptomatic COVID‐19 patients with ocular signs and/or symptoms are underreported. It is therefore important to inform patients and the public on potential ocular involvement, including signs and symptoms associated with COVID‐19 and to include questions about eye redness, itching and discharge in the clinical examination protocol at the time of acute presentation.

To date a limited number of studies on large animal models and ocular tissues explants have been performed and those have indicated that purposeful inoculation with a high viral SARS‐CoV‐2 load results in infection of the conjunctiva with ensuing mild respiratory tract symptoms. In the last few months, two contradictory studies have been published with the first using ocular tissue from 10 COVID‐19 deceased individuals, showing that corneal and conjunctival epithelium as well as the vitreous can be infected by SARS‐CoV‐2^74^ and a second study using cells expanded from the ocular surface epithelium showing that corneas are refractory to infection.^81^ This illustrates that this field of research is still in the early stage, with multiple groups relying on different but limited tissue sources to fully understand how SARS‐CoV‐2 enters, replicates and/or productively infects the ocular surface. The response of ocular surface to SARS‐CoV‐2 infection and its mounting immune response needs to be studied in detail, as important insights, which can be translated into prophylactic treatments may be gained. Last but not least, the role of tears in viral binding and transmission needs to be investigated as lactoferrin, a multifunctional protein implicated in the innate immune response, which is highly present in tears, has been shown to prevent SARS‐CoV binding to heparan sulfate proteoglycans,^70^ providing a potential prophylactic solution for exposed surfaces such as the ocular, buccal and nasal epithelium.

It is also important not to forget the short amount of time elapsed since the start of the COVID‐19 pandemic in December 2019, during which time it has not been possible to gather sufficient information from COVID‐19 positive infected patients and in vitro experimental studies to completely understand the role of the eye as part of the viral transmission dynamics, including penetration in the host cells, intracellular replication and the ocular surface immune response of infected individuals. Despite this as of 17th of December 2020, 84 140 peer‐reviewed manuscripts have been published, indicating a fast and impressive response by the scientific community to gain insights into viral mode of entry and impact on multiple organ systems. Importantly, coordinated efforts by large consortia and societies (such as Human Cell Atlas, UK Coronavirus Immunology Consortium, International Society for Stem Cell Research, etc) are enabling data sharing and exchange of scientific information at unprecedented rate to enable development of vaccines, cell and drug based therapies to win the arduous fight against the COVID‐19 pandemic. Importantly, increasing our understanding of the pathological mechanisms and modes of transmission of the SARS‐CoV‐2 virus will assist the necessary preparation to combat other viral diseases of this type and deliver prophylactic solutions to combat future pandemics more effectively.

## CONFLICT OF INTEREST

The authors declared no potential conflicts of interest.

## AUTHOR CONTRIBUTIONS

L.A. and M.L.: conceptualization, funding acquisition, writing—writing original draft, writing—review and editing; J.C.: funding acquisition, writing—writing original draft, writing—review; I.M.: writing—original draft; R.Q.: funding acquisition, writing—original draft; F.C.F.: conceptualization, funding acquisition, writing—writing original draft, writing—review.

## Supporting information


**Table S1** Summary of clinical findings of ocular surface involvement in COVID‐19.

**Table S2** The expression of the primary entry factors of SARS‐CoV‐2, ACE2 and TMPRSS2, in the different organs of the body. X refers to the factor's presence detected either at the transcript or protein level. The absence of an X may not necessarily be due to absence but due to the need for further research.

## Data Availability

Data sharing is not applicable to this article as no new data were created or analyzed in this study.
