# Supplementary material for: In the Eye of the Storm: SARS-CoV-2 Infection and Replication at the Ocular Surface?
Source: Stem Cells Transl Med. 2021 Mar 12;10(7):976–86. doi: 10.1002/sctm.20-0543 (PMC8235146; doi:10.1002/sctm.20-0543)
Supplement: stcltm312921-sup-0001-TableS1 — Table S1 Summary of clinical findings of ocular surface involvement in COVID-19. [file stcltm312921-sup-0001-tables1.docx]

| **Study type and region** | **Patient number** | **Tissue tested for viral presence** | **Viral detection method** | **Viral presence in ocular tissue** | **Ocular manifestations and findings** | **Other observations** | **Clinical examination methods** | **Reference** |
| --- | --- | --- | --- | --- | --- | --- | --- | --- |
| Retrospective study, Hubei Province, China | 38 in total: 28 were positive on RT-PCR screens of nasopharyngeal swabs | Nasopharyngeal and conjunctival swabs | RT-PCR | 2/28 (5.2%) yielded positive results on RT-PCR screens of conjunctival swabs | 12/38 patients had ocular manifestations consistent with conjunctivitis and of these, 11 were positive on RT-PCR screens of nasopharyngeal swabs | Patients with ocular symptoms were more likely to have higher white blood cell and neutrophil counts and higher expression of lactate dehydrogenase and C reactive protein than patients without ocular syndromes | Chest computed tomographic scans, blood tests, temperature measurements and ocular examination | [1] |
| Retrospective study, Wuhan, China | 33 COVID-19 patients without ocular manifestations | Nasopharyngeal and ocular surface swabs: 74.8% of these were collected more than 1 week after the onset of the disease | RT-PCR | 2/33 (6%) yielded positive results on RT-PCR screens of ocular surface swabs | No ocular manifestations reported | Viral RNA was detected in the ocular surface of 2 COVID-19 patients without conjunctivitis. The ocular samples of these patients were collected within 7 days of COVID-19 diagnosis | No data on clinical examination methods | [2] |
| Cross-sectional study, Madrid, Spain | 543 hospitalised patients. Recruitment criteria: over 18 years of age; patient with positive RT-PCR test from nasopharyngeal swab for SARS-CoV-2, hospitalized due to COVID-19, conjunctivitis diagnosis and ability to give verbal consent | Nasopharyngeal and conjunctival swabs | RT-PCR | 28/543 were identified with possible conjunctivitis and 21 of these yielded positive results on RT-PCR screens of nasopharyngeal swabs. Of these 14 were finally diagnosed with conjunctivitis and conjunctival swab was collected | SARS-CoV-2 RNA was detected in conjunctival swab of one patient (7%) among the 14 patients with conjunctivitis and laboratory-confirmed COVID-19 | Most conjunctival swabs were taken on the second day of conjunctival symptoms | Upon notification of possible conjunctivis, ocular examination and collection of a conjunctival swab was performed by two ophthalmologists | [3] |
| Retrospective study of 1099 patients with laboratory-confirmed Covid-19 from 552 hospitals in 30 provinces, autonomous regions, and municipalities in mainland China through January 2020: 43.9% of these were residents of Wuhan Province | 1099 COVID-19 patients | Nasopharyngeal swabs | RT-PCR | No tests were performed on ocular surface swabs | 9/1099 (0.8%) displayed conjunctival congestion | 926 were categorised as non-severe and 173 severe. Elevated levels of C-reactive protein were present in most patients. Patients with severe disease had more prominent lymphocytopenia and leukopenia than those with non-severe disease | Chest radiography or computed tomography, blood tests, temperature measurements | [4] |
| Prospective interventional case series study of 30 confirmed novel coronavirus pneumonia from the First Affiliated Hospital of Zhejiang University, China from 26 January 2020 to 9 February 2020 | 30 COVID-19 patients | Tear and conjunctival secretions collected twice | RT-PCR | Only 1/30 (3.3. %) of COVID-19 patients yielded positive results on RT-PCR screens of conjunctival secretions and tear fluids. | No ocular manifestations  reported | Viral RNA was detected in samples taken in the early course of the disease (3 days) | Computerised tomography lung imaging, temperature measurement and collection of a sputum sample. The first collection of tears and conjunctival secretions occurred within 7.33 ± 3.82 of disease onset | [5] |
| Cross-sectional study of patients who received a Covid-19 diagnosis, from 30^th^ December 2019 to 7^th^ February 2020 at Tongji hospital, Tongji medical college, Huazhong University of Science and Technology, China | 102 patients: 72 positive for SARS-CoV-2 on RT-PCR screens of nasopharyngeal swabs | Nasopharyngeal and conjunctival swabs | RT-PCR | 2/72 patients (2.78%) had conjunctivitis. Only 1 out the 2 patients with conjunctivitis yielded positive result on RT-PCR tests of conjunctival swabs taken on the 3^rd^ day of disease onset. | Conjunctivitis reported in 2 patients | The ocular examination of patient with confirmed viral presence in conjunctival sample revealed conjunctival congestion and watery discharges in both eyes with normal best corrected visual acuity, normal corneal epithelium, quiescent anterior chamber and no tenderness or enlargement of the preauricular lymph node | Computerised tomography lung imaging, temperature measurement and blood tests.  The first collection of conjunctival samples occurred within 18.15 ± 7.57 of disease onset | [6] |
| Retrospective study of 67 confirmed or suspected COVID-19 patient during 17-28 January 2020, Hubei Province, China | 67 patients: of those 63 were identified as laboratory confirmed COVID-19 cases | Nasopharyngeal and conjunctival swabs | RT-PCR | 1/67 (1.4%) of the patients yielded positive findings on RT-PCR in conjunctival swab samples.  2/67 yielded probable positive results. | One patient with conjunctivitis had negative findings by RT-PCR. The patient had a history of contact with a confirmed COVID-19 patient wearing a surgical mask but no protective goggles | There was variation in ocular symptoms between patients, but in general these were mild and tended to be self-healing. In the early stages, it appeared as common conjunctival hyperemia with fewer secretions. Occasionally small pieces of conjunctival haemorrhage were observed |  | [7] |
| Case study | 1 confirmed COVID 19 patient | Nasopharyngeal and conjunctival swabs | RT-PCR | The conjunctival swabs yielded positive results from day 9-18 of the disease. | On day 8, the patient reported redness, foreign body sensation and tearing in both eyes without blurred vision. Acute viral conjunctival infection was confirmed through slit lamp examination. By day 14 all ocular symptoms had resolved | Ocular symptoms observed in the middle phase of the disease | Slit lamp examination, collection of saliva and sputum samples, lung auscultation | [8] |
| First Aﬃliated Hospital of Zhe-  jiang University  First Aﬃliated Hospital of Zhe-  jiang University  Retrospective study in the First Affiliated Hospital of Zhejiang University, China | 56 COVID-19 confirmed patients |  |  | 15 subjects (27%) reported ocular symptoms in including sore eyes, itching, foreign body sensation, tearing, redness, dry eyes, eye secretions and floaters. Six of these patients presented with ocular symptoms before onset of fever or respiratory symptoms. Two of the six subjects developed conjunctivitis. Viral RNA was detected in conjunctival swabs of one of the subjects with conjunctivitis | Four subjects reported the appearance of ocular symptoms 1 to 7 days before onset of fever or respiratory symptoms; however, no viral RNA was found in their tear or conjunctiva samples |  | Medical history including exposure and measures taken for personal protection was reviewed together with ocular history | [9] |
| Prospective study, Singapore | 17 COVID-19 patients | Nasopharyngeal swabs |  |  | None of the 17 patients recruited demonstrated ocular symptoms. 1 patient developed conjunctival injection and chemosis. Fourteen patients showed upper respiratory tract symptoms at presentation | Tear samples collected on week 1-3 of disease onset | Clinical data, including age, gender, symptoms, and ocular symptoms including red eye, tearing, blurring of vision, discharge, and colour desaturation were collected | [10] |
| Bambino Gesù Children's Hospital  Prospective observational case series study in Bambino Gesù Children's Hospital | 27 paediatric patients with confirmed COVID-19 | Nasopharyngeal and conjunctival swabs | 4/27 (15%) of patients presented with ocular manifestation characterized by mild conjunctival hyperemia and secretion. 3 patients (11%), 1 symptomatic and 2 asymptomatic had positive findings for COVID-19 on conjunctival swabs |  | Clinical resolution of conjunctivitis was achieved in all patients 3-5 days after onset | Swabs were repeated 4 times on average | Clinical data, including demographic information, contact history, clinical symptoms, and laboratory findings were reviewed and analysed. Ocular symptoms and signs were also recorded | [11] |
| Case series study in Renmin Hospital of Wuhan University, China Case series study in Renmin Hospital of Wuhan University, China Case series study in Renmin Hospital of Wuhan University, China  Case series study in Renmin Hospital of Wuhan University, China | 121 COVID-19 patients | Nasopharyngeal and conjunctival swabs collected on the same day | RT-PCR | Only 3/121 (2.5%) COVID-19 patients yielded positive results on RT-PCR screens of conjunctival swabs | 52.1 % had mild or moderate disease and 47.9% has severe or critical disease. 8/121 (6.6%) showed ocular symptoms, however only 1 of these 8 patients yielded positive results on the conjunctival swab | The ocular symptoms included itching, redness, tearing, discharge, and foreign body sensation. The conjunctival samples were obtained from one of the affected eyes of patients with ocular symptoms or randomly from 1 eye of patients without ocular symptoms | Relevant clinical information and any ocular symptoms that developed either at onset or during the later course of the disease were obtained through the review of medical records and implementation of the external eye examination with a pen light | [12] |
| Online questionnaire | 83 participants with confirmed COVID-19 |  |  |  |  | The most common reported ocular symptoms were:  photophobia (18%), sore eyes (16%) and itchy eyes (17%) | 81% reported eye symptoms within two weeks of other COVID-19 symptoms. No differences in eye related problems were observed between males and females | [13] |
| Systematic review and meta-analysis | 16 studies reporting 2347 confirmed COVID-19 cases | 6/16 studies reported data on RT-PCR positivity from conjunctival swabs or tear samples of COVID-19 patients with or without ocular symptoms |  | Viral RNA was detected in the ocular specimens of 3.5% patients. |  | Ocular manifestations observed in 11.64% of COVID-19 patients. Main ocular features were ocular pain (31.2%), discharge (19.2%), redness (10.8%), and follicular conjunctivitis (7.7%) 6.9% patients with ocular manifestations had severe pneumonia | The available studies show significant publication bias and heterogeneity | [14] |
| Systematic review and meta-analysis | 14 studies |  |  | The pooled detection rate of virus in conjunctiva was 1% |  | Ocular manifestations amongst COVID-19 patients estimated at 6-32%, with pooled prevalence estimated as 7%. The most common symptoms included conjunctival hyperemia, increased secretion, pain, and foreign body sensation |  | [15] |

**Table S1:** **Summary of clinical findings of ocular surface involvement in COVID-19.**

**References**

1 Wu P, Duan F, Luo C, et al. Characteristics of Ocular Findings of Patients with Coronavirus Disease 2019 (COVID-19) in Hubei Province, China. JAMA Ophthalmol 2020;138:575–578.

2 Xie H-T, Jiang S-Y, Xu K-K, et al. SARS-CoV-2 in the ocular surface of COVID-19 patients. Eye Vis 2020;7:23.

3 Güemes-Villahoz N, Burgos-Blasco B, Arribi-Vilela A, et al. SARS-CoV-2 RNA detection in tears and conjunctival secretions of COVID-19 patients with conjunctivitis. J Infect 2020;81:452–482.

4 Guan W, Ni Z, Hu Y, et al. Clinical Characteristics of Coronavirus Disease 2019 in China. N Engl J Med 2020;382:1708–1720.

5 Xia J, Tong J, Liu M, et al. Evaluation of coronavirus in tears and conjunctival secretions of patients with SARS-CoV-2 infection. J Med Virol 2020;92:589–594.

6 Zhang X, Chen X, Chen L, Deng C, Zou X, Liu W, et al. The evidence of SARS-CoV-2 infection on ocular surface. Ocul Surf. 2020;18:360–2.

7 Zhou Y, Zeng Y, Tong Y, et al. Title: Ophthalmologic evidence against the interpersonal transmission of 2019 novel coronavirus through conjunctiva. MedRxiv 2020:2020.02.11.20021956.

8 Chen L, Liu M, Zhang Z, et al. Ocular manifestations of a hospitalised patient with confirmed 2019 novel coronavirus disease. Br J Ophthalmol 2020;104:748–751.

9. Hong N, Yu W, Xia J, Shen Y, Yap M, Han W. Evaluation of ocular symptoms and tropism of SARS-CoV-2 in patients confirmed with COVID-19. Acta Ophthalmol. 2020: 98: e649–e655.

10. Seah IY, Anderson DE, Kang AEZ, Wang L, Young BE, Lye DC, Agrawal R. Assessing Viral Shedding andInfectivity of Tears inCoronavirus Disease 2019(COVID-19) Patients. [Ophthalmology](https://www.ncbi.nlm.nih.gov/pmc/articles/PMC7151491/). 2020 Jul; 127(7): 977–979.

11. Valente P, Giancarlo R, Federici M, Petroni S, Palma P, Cotugno M, Ioris MA, Campana A and Buzzonetti M. Ocular manifestations and viral shedding in tears of pediatric patients with coronavirus disease 2019: a preliminary report. [Journal of American Association for Pediatric Ophthalmology and Strabismus](https://www.sciencedirect.com/science/journal/10918531) 2020; 4: 212-215.

12. Zhou Y, Duan C, Zeng Y, Tong Y, Nie Y, Yang Y, et al. Ocular findings and proportion with conjunctival SARS-COV-2 in COVID-19 patients. Ophthalmology. 2020;127:982–3.

13. Pardhan S, Vaughan M, Zhang J, Smith L and Chichger H. Sore eyes are the most significant ocular symptoms experienced by people with COVID-19:a comparison between pre-COVID1 and during COVID-19 states. BMJ Open Opthalmology 2020: 5: e000632.

14. Aggarwal K, Aggarwal N, Jaiswal N, Dahiya N, Ahuja A, Mahajan A, Tong L, Duggal M, Singh M, Aggraval R and Gupta M. Ocular surface manifestations of coronavirus disease 2019 (COVID-19): A systematic review and meta-analysis. PLoS One. 2020 Nov 5;15(11):e0241661.

15. Ling XC, Kang, EYC, Lin JY, Chen HC, Lai CC, Ma DHK, Wu WC. Ocular manifestation, comorbidities, and detection of severe acute respiratory syndrome-coronavirus 2 from conjunctiva in coronavirus disease 2019: A systematic review and meta-analysis. Taiwan Journal of Ophthalmology 2020; 10: 153-166.
