# Supplementary material for: In the Eye of the Storm: SARS-CoV-2 Infection and Replication at the Ocular Surface?
Source: Stem Cells Transl Med. 2021 Mar 12;10(7):976–86. doi: 10.1002/sctm.20-0543 (PMC8235146; doi:10.1002/sctm.20-0543)
Supplement: stcltm312921-sup-0002-TableS2 — Table S2 The expression of the primary entry factors of SARS-CoV-2, ACE2 and TMPRSS2, in the different organs of the body. X refers to the factor's presence detected either at the transcript or protein level. The absence of an X may not necessarily be due to absence but due to the need for further research. [file stcltm312921-sup-0002-tables2.docx]

**Table S2: The expression of the primary entry factors of SARS-CoV-2, ACE2 and TMPRSS2, in the different organs of the body.** X refers to the factor’s presence detected either at the transcript or protein level. The absence of an X may not necessarily be due to absence but due to the need for further research.

| **Location** | **SARS-CoV-2 Entry Factor** | | **Reference** |
| --- | --- | --- | --- |
|  | **ACE2** | **TMPRSS2** |  |
| Aqueous humour | X |  | [1] |
| Conjunctival epithelium | X | X | [2–4] |
| Limbal epithelium | X | X | [2,4] |
| Corneal epithelium | X | X | [2,4,5] |
| Nasal epithelium | X | X | [2,5,6] |
| Oral mucosa | X |  | [7] |
| Salivary gland |  | X | [8] |
| Oesophagus | X | X | [5,9] |
| Upper airways and lungs | X | X | [5,8] |
| Heart | X | X | [5] |
| Liver | X | X | [5,6,8] |
| Kidney | X | X | [5,8] |
| Pancreas |  | X | [5,8] |
| Stomach | X | X | [6,8] |
| Prostate |  | X | [5,8] |
| Colon | X | X | [5,8] |
| Small intestine | X | X | [5,6,8] |
| Gall bladder | X | X | [5] |
| Bile Duct | X | X | [5,8] |
| Testis | X | X | [5,8,10] |
| Ovaries |  | X | [8] |
| Rectum | X |  | [9] |

**References**

1 Holappa M, Valjakka J, Vaajanen A. Angiotensin(1-7) and ACE2, “The Hot Spots” of Renin-Angiotensin System, Detected in the Human Aqueous Humor. Open Ophthalmol J 2015;9:28–32.

2 Collin J, Queen R, Zerti D, et al. Co-expression of SARS-CoV-2 entry genes in the superficial adult human conjunctival, limbal and corneal epithelium suggests an additional route of entry via the ocular surface. Ocul Surf 2020.

3 Ma D, Chen CB, Jhanji V, et al. Expression of SARS-CoV-2 receptor ACE2 and TMPRSS2 in human primary conjunctival and pterygium cell lines and in mouse cornea. Eye 2020;34:1212–1219.

4 Zhou L, Xu Z, Castiglione GM, et al. ACE2 and TMPRSS2 are expressed on the human ocular surface, suggesting susceptibility to SARS-CoV-2 infection. Ocul Surf 2020;18:537–544.

5 Sungnak W, Huang N, Bécavin C, et al. SARS-CoV-2 entry factors are highly expressed in nasal epithelial cells together with innate immune genes. Nat Med 2020;26:681–687.

6 Qi F, Qian S, Zhang S, et al. Single cell RNA sequencing of 13 human tissues identify cell types and receptors of human coronaviruses. Biochem Biophys Res Commun 2020;526:135–140.

7 Xu H, Zhong L, Deng J, et al. High expression of ACE2 receptor of 2019-nCoV on the epithelial cells of oral mucosa. Int J Oral Sci 2020;12:8.

8 Bugge TH, Antalis TM, Wu Q. Type II transmembrane serine proteases. J Biol Chem 2009;284:23177–23181.

9 Qi F, Qian S, Zhang S, et al. Single cell RNA sequencing of 13 human tissues identify cell types and receptors of human coronaviruses. Biochem Biophys Res Commun 2020;526:135–140.

10 Sama IE, Ravera A, Santema BT, et al. Circulating plasma concentrations of angiotensin-converting enzyme 2 in men and women with heart failure and effects of renin–angiotensin–aldosterone inhibitors. Eur Heart J 2020;41:1810–1817.
